# Progress in Cerebral Surgery

**Published:** 1901-12-21

**Authors:** 


### Progress in Cerebral Surgery.

Several writers have contributed towards the surgical treatment of trifacial neuralgia. The excision of the Gasserian ganglion finds not a few advocates, and successful cases have been reported. Two of these were in the hands of Willard Bartlett,[1] who excised the ganglion intact, and so procured two excellent specimens for microscopic examination. He used Cushing's inferior temporal method, which aims at avoiding the middle meningeal artery by making the opening low in the temporal fossa. In his first case he was met by profuse venous hæmorrhage on separating the dura mater from the bony floor of the mid-cranial fossa. This was successfully arrested by means of packing, and the second and third branches were cut, and the ganglion evulsed, with part of its sensory root, being torn away from its first branch. A rather intractable corneal ulcer developed and delayed convalescence, but after four months this was well, and the patient entirely without pain. His second case was more straightforward. There was no hæmorrhage during the operation requiring packing, and the convalescence was not delayed by trophic changes. Some motor disturbances resulted in both cases, but were temporary only. Pain was entirely relieved.

The dangers of this operation are well brought out in an article by Robert Amyx,[2] who gives a detailed account of the relations of the ganglion to its surrounding structures, and also methods to be adopted to avoid injuring the important blood-vessels and nerves. This article is the outcome of a series of operations upon over 50 skulls. He divides skulls into three varieties for this operation: (*a*) those which are broad in front of the ears, which show no bony prominences arising from the floor of the middle fossa; (*b*) those that are narrow in this measurement, which present bony prominences, which cause considerable difficulty in operating; (*c*) and those which present a combination of these two. He emphasises the importance of an accurate knowledge of the attachments of the dura mater, and recommends (1) that careful dissection of the ganglion be made from the dura and other contiguous structures, always working towards the ganglion or its branches, to avoid injury to the surrounding structures; (2) that the ganglion should be carefully and completely freed from its dural envelope, as well as from the internal carotid and middle meningeal arteries and cavernous sinus. He would then divide the second and third branches and break through the trunk of the ganglion, pulling it away from the cavernous sinus before attempting to cut the first branch. This avoids injury to the sixth nerve and the contents of the cavernous sinus. He holds that all hæmorrhage is easily controlled by packing, except in old patients and those with marked arteriosclerosis. Rather than run the risk of tearing important structures, Dr. Frazier[3] recommends the severance of the posterior roots only, holding that this effectually prevents re-establishment of function in the nerves of dogs.

Willard's ganglions were carefully examined by Schwab,[4] who endeavours to summarise the findings of the various reporters upon the pathological condition of 20 collected cases of removal of Gasserian ganglions. He claims that the variety of these findings, and even contradictions, make this endeavour difficult; but he divides them into sections thus: (1) One group where the ganglion is apparently normal. (2) A second where there is pronounced evidence of neuritis and degeneration. (3) A third, including one case of Spiller's and one of his own, showing an interstitial inflammation. (4) A large group showing nerve-cell changes of various degrees of severity. These changes seemed less primary than secondary. Especial mention is made of the presence of brain-sand or concretions and changes in the blood-vessels. He warns against the conclusions derived from ganglia which have been morcellated, and also in those specimens removed after peripheral operations have previously been performed. He stained his own specimens with the Nissl stain. In one of the two specimens removed entire he found that the smaller nerve-cells stained most deeply and showed the greatest abnormality; and he quotes Head and Campbell as to their finding that the smaller cells were first and most profoundly affected in the spinal ganglia in herpes zoster. He failed to find in either definite changes which are found in acute or long-standing parenchymatous nerve-cell affections; and he concludes from his own experience and the reports of others that "it is quite evident that trigeminal neuralgia is not a definite disease, but merely the symptom of various processes affecting the fifth nerve anywhere in its course, from the ganglion to its peripheral termination." He holds that there is justification for two main divisions of trigeminal neuralgic affections: (1) A neuritis beginning in the terminal divisions of the fifth nerve, and having a tendency to ascend to the ganglion—the commoner variety; and (2) An interstitial inflammation, chronic and progressive, of the ganglion body itself. A third division is possible, of an affection of the sensory root on its way from the ganglion to the pons.

A case suggestive of the localisation of the visual area in the occipital cortex is cited by Sanders.[5] The patient was a soldier in the 1st Liverpool Regiment who received a bullet in the back of the head, which wounded the brain under a spot the centre of which was 1½ inch from the median line and 2½ inches above the right occipital protuberance. The immediate symptom was blindness, but whether total or not could not be ascertained, and ten days later, on the journey from the front to the base hospital, he grew worse and lost consciousness. The limbs were extended, breathing deep, eyes closed, if aroused became noisy and abusive; temperature was 103·5, pulse 76, with sharp beat and increased tension. The wound was found to be suppurating, and the pus was released and the wound dressed. On this the temperature fell, but the impairment of sight remained great; five days after the dressing he only barely distinguished the sister and the colour of her dress. Four days later he was found to have marked diminution of both fields of vision; and a month later it was found by means of a perimeter that vision was limited to a small area round each of the fixation-points, with no marked difference in the eyes, and he was unable to read half-inch type further away than two feet. No evidence of mind blindness.

The writer draws attention to the unilateral position of the injury: he does not consider the opposite side to have been injured, the bullet having evidently entered and left by the same side. On marking out the same position on a skull, he came to the conclusion that the parts of the brain injured were (1) the anterior part of the occipital cortex on its outer aspect; (2) the hindmost portion of the angular gyrus; (3) part of the white substance of the occipital lobe, including part of the optic radiation, and the white substance underlying somewhat deeply the angular gyrus. He concludes that in many both sides of both retinæ are represented to some extent on one side of the brain. That both macular regions should have escaped, partially at any rate, may be explained by the bilateral representation of portions of both maculæ. He considers that this case supports the opinion of those who regard the angular gyrus as part of the visual cortex; and describes the case as one of left hemianopia due to the damage of the occipital lobe, with concentric contraction of the fields of vision on the same side as the lesion, due to the destruction of fibres of the optic radiation beneath the angular gyrus and to the injury to that convolution."

The surgery of abscess in the brain is well reviewed by C. A. Ballance,[6] who points out that these collections of pus may remain latent for some time and be awakened into life by some trivial accident or operation. The brain, he says, should be treated like any other part of the body, in that abscesses should be freely opened and drained. For the purpose of reaching the pus he recommends the use of the sharp-pointed, long, and narrow knife, rather than of the trocar and cannula. The disadvantages of the latter are that the abscess may be missed, or be transfixed without being drained, or that the capsule of an old collection may be too tough to be pierced. If the trocar and cannula be used in the cerebellum, it should be made of platinised silver and have rings in the cannula which shall serve to maintain it in position once pus has been reached, the danger being that it may be accidentally withdrawn and the abscess not found again. Recurrence of the symptoms may be due to failure in the drainage or to a fresh abscess being formed in the same lobe. Especially may this be the case in the cerebellum, where a succession of fresh abscesses is not uncommon. This fresh formation may produce symptoms leading to a diagnosis of meningitis. He recommends gold leaf as an efficient protective to a hernia cerebri, which should be treated by elastic pressure. That an abscess, even though extensive, may be present without causing symptoms for some time is well instanced by a case reported by Trevor Jones.[7] A man had a blow on the head which produced some scalp injuries. For 25 days the patient seemed in excellent health. After a drunken bout he became aphasic and one scalp wound was found septic, a few beads of pus being released. Next day his right arm and leg were paralysed. Coma ensued on the following day, with constant hiccough, absolute right-sided hemiplegia, subnormal temperature, stertor, and respiration 55 per minute. The discharges were passed under him. No depressed fracture was found, but on raising a scalp flap and periosteum a fissured fracture was revealed. On trephining over this, the trocar was plunged into the frontal lobe, and pus came away freely. The cavity was explored with the finger and found to extend backwards as far as could be reached. The cavity was flushed with 1 in 40 carbolic-acid lotion, and a drainage tube left in; 8,000 units of anti-streptococcin were given. As improvement was not apparent the wound was reopened to allow of freer drainage. Aphasia disappeared and recovery was complete; the patient was discharged well three months later. It is interesting to remark that, in dressing the wound, if a certain part of the frontal lobe were touched, hiccough immediately resulted.

Dawbarn [8] records a case of epidural hæmorrhage which gave the symptoms of right-sided pressure. On operating no blood was found, but at subsequent post-mortem a large clot was found on the same side as the paralysis, and microscopically the fibres were found not to have decussated. There were only 20 of such cases recorded. According to Henry Wharton's collection of 70 injuries to venous sinuses within the skull,[9] the longitudinal sinus was the most often injured, then the lateral sinus, and the cavernous, straight, and transverse sinuses were but rarely damaged. Prescott Hewitt makes the lateral sinus the most frequently injured, while Agnew and Phelps agree with the author. The longitudinal sinus is most easily injured by direct, and the lateral by transmitted violence.

Wharton holds that (1) wounds of venous sinuses of the brain should be classed as dangerous injuries, being followed by a high mortality from extra or intra-cranial hæmorrhage, or especially from septic infection; (2) such wounds should be packed with gauze to control hæmorrhage, as the most satisfactory method. Ligation is only available in some and not at all in other cases. Lateral ligature may be applied in a few cases, and is less dangerous than total ligature; and sutures can be well employed in small wounds which are freely exposed. He holds that pressure by forceps is a ready but dangerous method and has no advantage over other means.

In operating to relieve a case of traumatic epilepsy Keen [10] raised a wide flap of thinned cortex under the belief that it was the wall of a cyst, and found that he had opened the lateral sinus; the fornix, choroid pexus, and cornua were recognised. A large amount of cerebro-spinal fluid escaped without harm; indeed, on unplugging the wound and letting more fluid escape headache was relieved. The epilepsy was not cured by the operation, but the patient left the hospital no worse for the treatment.

Three cases of fracture-dislocation of the spine have been treated by laminectomy by Herbert Allingham.[11] Although two cases ended fatlaly, their end was not apparently hastened by the operation. One case recovered completely, and one of the fatal cases survived the operation 15 days, and died 33 days after the injury, with marked improvement in the paralysis and anæsthesia; unfortunately atony of the bowel and intractable diarrhœa terminated the case. With reference to such cases it is urged by Mummery,[12] and in this he is supported by McCosh,[13] that cases should be treated by laminectomy as soon as the injury or tumour can be located. McCosh warns against relying upon prolonged treatment by "antisyphilitics," as allowing of the degenerative processes to proceed so far as to make the result hopeless. Perhaps earlier operation might have been of advantage in an interesting case cited by Sailer,[14] where a man had his cord injured by a Mauser small-calibre bullet. The wound was immediately followed by intense pain and loss of sensation in the lower part of the body and complete paraplegia (November 2nd, 1896). Atrophy of the lower limbs and bedsores over sacrum, buttocks, and ankles resulted, with paroxysmal pains in the thighs and lower abdomen. In May, 1898, he showed some returning power in the left hip and knee, with exaggerated knee-jerk, but no ankle-clonus or plantar-reflex. Slight flexion in the right thigh was due to contraction of ilio psoas, and slight power of adduction was present; no reflexes obtainable, and anæsthesia below upper third of thigh and a three-inch band of hyperæsthesia encircled the limb. Total anæsthesia in left leg. In November, 1899, lightning pains in the thighs and lower abdomen were not relieved by drugs, so laminectomy was performed over the 10th, 11th, 12th dorsal, and 1st lumbar vertebræ. The dura mater was found greatly thickened with adhesions between it and the pia mater; the cord was apparently normal. After four weeks there was improvement in the hips and knees, but none in the ankles or toes. The pains were worse during these four weeks. Total anæsthesia remained over the sacral area and partial anæsthesia in perineum. Allochiria was noticed 39 days after operation, and persisted for three days only.

[1] Annals of Surgery, June. [2] Med. Record, July 6. [3] Med. Record, June 8. [4] Annals of Surgery, June. [5] Lancet, Aug. 31. [6] Lancet, May 25. [7] Brit. Med. Jour., June 1. [8] Med. Record, June 8. [9] Annals of Surgery, July. [10] Amer. Jour. Med. Sc., July. [11] Lancet, May 18. [12] Ibidem. [13] Med. Record, June 8. [14] Lancet, June 13.

